# Characteristics of multi-channel intermuscular directional coupling based on time-varying partial directional coherence analysis

**DOI:** 10.1038/s41598-023-43976-0

**Published:** 2023-10-10

**Authors:** Yihao Du, Qiang Fan, Chaoqun Chang, Xiaolin Bai, Tianfu Cao, Yanfu Zhang, Xiaoran Wang, Ping Xie

**Affiliations:** Key Lab of Measurement Technology and Instrumentation of Hebei Province, Institute of Electric Engineering, Yanshan University, Qinhuangdao, 066004 Hebei People’s Republic of China

**Keywords:** Biological techniques, Bioinformatics

## Abstract

The human body transmits directional information between muscles during upper limb movements, and this will be particularly evident when the dominant muscle changes during movement transitions. By capturing the electromyography (EMG) signals of wrist flexion and extension continuous transition movements, we investigated the characteristics of multichannel intermuscular directional coupling and directional information transmission, and consequently explored the control mechanism of Central nervous system (CNS) and the coordination mechanism of motor muscles. Multi-channel EMG was collected from 12 healthy subjects under continuous translational movements of wrist flexion and extension, and the time-varying biased directional coherence analysis (TVPDC) model was constructed using partial directional coherence analysis (PDC) frequency domain directionality to study the directional information transfer characteristics in the time–frequency domain, screen closely related muscle pairs and perform directional coupling significance analysis. Palmaris longus (PL) played a dominant role under wrist flexion movements(WF), Extensor Carpi Radialis (ECR) played a dominant role under wrist extension movements(WE), and the remaining muscles responded to them with information and Biceps Brachii (BB) played a responsive role throughout the movement; flexor pairs had the highest positive coupling values in the beta band during Conversion action1 (MC1) and WF phases, and extensor pairs had the highest positive coupling values in the gamma band during Conversion action2(MC2) phase and the highest coupling values in the beta band during WE phase. TVPDC can effectively analyze the multichannel intermuscular directional coupling and information transmission relationship of surface electromyography under wrist flexion and extension transition movements, providing a reference for exploring the control mechanism of CNS and abnormal control mechanism in patients with motor dysfunction in a new perspective.

## Introduction

The movement of the human upper limbs is a very complicated process under the influence of the Central Nervous System (CNS) which is the consequence of the joint action of movement-related muscle groups in a certain spatio-temporal relationship^1^.The neurological system exerts control over muscle groups through neural oscillations to achieve inter-muscular coordination, which not only reflects the coordinated control mechanism of the CNS, but also the feedback from muscle groups to the CNS. In addition, since the relevant muscles in upper limb movements act as synergistic and antagonistic muscles, correspondingly, the muscles act differently in different movement patterns and exhibit different activity states and functional relationships. Therefore, it is an essential theoretical reference and diagnostic physiological basis to analyze the directional information transmission relationship between muscles in upper limb motor transitions, and then to explore the motor control mechanism of CNS and the abnormal control mechanism in patients with motor dysfunction.

Due to the surface electromyography (sEMG) signal contains rich CNS control information and muscle activation information^2^, the activity states and functional relationships of muscle groups could be obtained by analyzing sEMG features from multiple perspectives. It has been suggested that the motion control system, in the process of innervating upper limb movements, activates the motor-related muscle groups as a whole to simplify the motor process^3^, while the muscles within the muscle groups are driven by a common corticospinal cord^4^, so there are different degrees of information interactions between the muscles, i.e., the phenomenon of intermuscular coupling. Thus, intermuscular coupling characteristics could reflect the intrinsic mechanism of the motor state and control mode of different muscles, e.g. intermuscular coupling in the alpha band is mainly under control of spinal nerves^5^. The intermuscular coupling in the beta band is associated with isometric muscle contractions and could reflect information transfer in the corticospinal pathway^6,7^; the coupling phenomenon in the gamma band is reflected in the integration of information associated with very vigorous muscle contractions and cognitive processes^8,9^, which are mainly controlled by the cortical nerves of the brain^10^. In addition, Russo et al.^11^ combined non-negative matrix decomposition and coherence analysis to explore the muscle synergistic-coupling relationship under wrist flexion and wrist extension movements, and more closely investigated the way in which CNS control mechanisms and muscles cooperate with each other, Tian et al.^12^ introduced transfer entropy and generalized biased directional coherence into the analysis of intermuscular coupling of wrist flexion movements in the flexed elbow state, and deeply revealed the CNS control mechanisms in the dominance of synergistic and antagonistic muscles, Hu et al.^13^ combined non-negative matrix decomposition and time–frequency coherence to explore the time-varying coherence between synergistic muscles in wrist movement sequences and demonstrated the time-varying mechanisms of synergistic modulation and synchronous oscillation in the CNS control mechanism, Lee et al.^14^ used a multi-scale time–frequency coupling analysis model to study the anterior bundle of deltoid muscle in healthy individuals and patients by constructing a multi-scale time–frequency coupling analysis model for The differences in the time–frequency coupling properties of the anterior, middle and posterior deltoid bundles in healthy subjects and patients were investigated by Lee et al. Most of these studies focused on the experimental paradigm of wrist flexion/extension, with a focus on the intermuscular coupling properties in the frequency or time–frequency domains, and did not consider the intermuscular coupling properties during the transition from wrist flexion/extension to wrist extension/flexion movements, and rarely investigated the directional information transfer relationships during movements. In addition, the consistency method used for intermuscular coupling analysis^15^ focuses on the coupling strength of two time series in the frequency domain, and Granger Causality (GC) analysis can only identify the directional information transfer between two time series, and cannot effectively analyse the directional information transfer relationship within a muscle group^16,17^

In summary, this paper designs an experimental paradigm for continuous translational movements of upper limb wrist flexion and extension, and investigates the directional coupling characteristics of multichannel sEMG between muscles using a Granger causality-based biased directional coherence analysis method; the model of time-varying biased directional coherence analysis was constructed to further validate and supplement the directional information transfer relationship under continuous wrist flexion–extension transition movements, and to filter the muscle pairs closely related to wrist flexion–extension movements; muscle pair directional coupling significance analysis was performed, and the results of 12 subjects were statistically analyzed to investigate the change pattern of directional coupling characteristics under continuous wrist flexion–extension transition movements, and then to investigate the motor control mechanism of CNS and the abnormal control mechanism of patients with motor dysfunction, so as to provide quantitative motor function status assessment and diagnostic reference for rehabilitation physicians.

## Results

In order to verify the feasibility of TV-PDC model, PDC analysis was performed on the collected sEMG data based on the wrist flexion and extension test, and further time frequency domain directional coupling analysis was performed using TVPDC model to verify and supplement the single time domain or frequency domain PDC results, while the muscle pairs with strong correlation with wrist flexion and extension movements were screened for directional coupling significance analysis, and finally the results were statistically analyzed to study the overall change pattern.

### Analysis of PDC results

**Figure 1:**
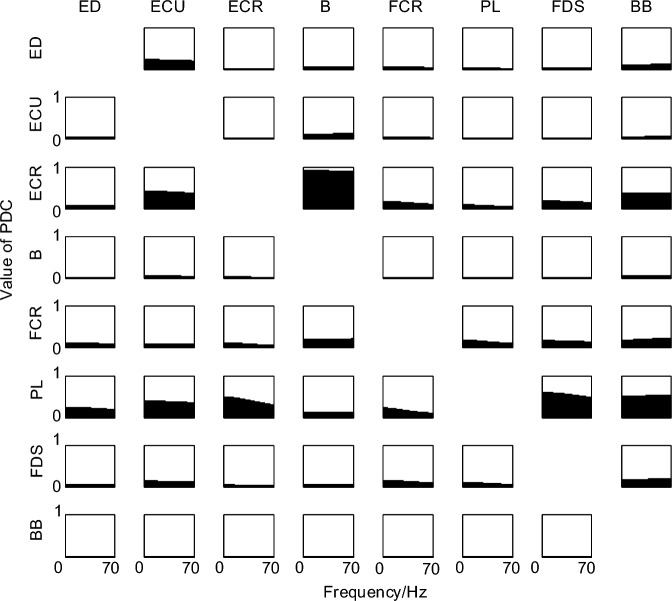
Results of 8-channel intermuscular PDC analysis.

The pre-treated sEMG data were analyzed using PDC and the mean PDC values were calculated for each subject for 10 replicate trials. Figure 1 shows the results of the analysis for a randomly selected subject, with the horizontal coordinate indicating the frequency and the vertical coordinate indicating the normalized PDC value. The shaded part of the plot in the -th row and -th column indicates the intensity of the information flowing from channel to channel, i.e., the magnitude of the PDC value in the -direction.

As seen in Fig. 1, the coupling intensity in the direction of ECR→B is the largest under the local angle, followed by PL→FDS and PL→BB, and the coupling intensity in the direction of ECR→ECU, ECR→BB and PL→ECR is also more obvious; the overall angle has the most information transmitted from ECR and PL to other muscles, followed by FCR, and there is also weak information transmitted from ED and FDS; conversely, the remaining muscles had almost no information transmission to the above muscles, and BB was influenced by almost all the remaining muscles, but BB had no information transmission to all the other muscles.

### Analysis of TVPDC results

**Figure 2:**
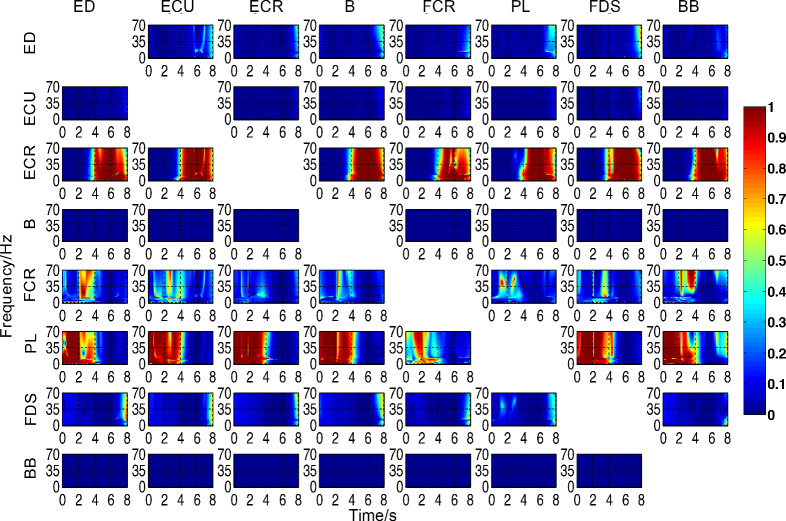
Results of 8-channel intermuscular TVPDC analysis.

Moreover, TVPDC was used to perform the analysis of the sEMG data under continuous wrist flexion and extension transitions, while the results of the data segment under the relaxed state in the test movement were removed for the convenience of observation, and only the 8-s analysis results under continuous movements (MC1→WF→MC2→WE) were studied. Figure 2 shows the results of TVPDC analysis for the same subject, the horizontal coordinates indicate time, the vertical coordinates indicate frequency, and different colors in the figure indicate the magnitude of TVPDC values.

As seen in Fig. 2, the most information was transmitted from ECR and PL to other muscles under the overall perspective, followed by FCR, with very weak information coming out from ED and FDS, and no significant information transmission from the remaining muscles, which is basically consistent with the results of PDC analysis; the information transmitted from ECR to all the remaining muscles under the local perspective occurred mainly in MC2 stage and WE stage, the information transmitted from PL to all the remaining muscles occurred in MC1 and WF stage, while FCR had information transmitted to the remaining muscles in both WF stage, but the degree of its influence was smaller than PL and ECR, and ED and FDS had weak information transmitted to the remaining muscles at the end of WE stage, and finally BB was Each phase was influenced by the remaining muscles, with ECR and PL having the greatest influence, fully demonstrating that the TVPDC model can effectively analyze the multichannel intermuscular directional information transfer relationship with time and frequency. In addition, compared to the results of TVPDC analysis in the horizontal coordinate—time domain, the results of TVPDC analysis in the vertical coordinate—frequency domain were less pronounced, but differences in TVPDC values could still be observed: the intensity of coupling between ECR and PL and the rest of the muscles decreased with increasing frequency, while the degree of influence of FCR on the rest of the muscles increased with increasing frequency.

### Exercise-related muscle pair screening

Integrating the results of PDC and TVPDC analyses in 12 subjects, the muscle pairs that can be screened to be closely related to the continuous transition movements of wrist flexion and extension are PL-ECR, PL-FDS, and PL-BB under wrist flexion movements and ECR-ECU, ECR-B, and ECR-BB under wrist extension movements^13^, as shown in Table 1.

**Table1:** Muscle pairs with larger coupling values at different exercise phases in 12 subjects.

Subjects	Flexor pair	Extensor pair
H1	PL → BB, FDS → BB, FDS → ECU	ECR → B, ECR → BB
H2	PL → FDS, PL → BB, FCR → BB	ECR → B, ECR → BB, ECR → ECU
H3	PL → BB, FDS → FCR	ECR → B, ECR → BB, ECR → PL
H4	PL → BB, FDS → BB, FCR → BB	ECR → B, ECR → BB, ECR → ECU
H5	FCR → BB, FDS → PL	ECR → B, ECR → ED, ECR → PL
H6	PL → FDS, PL → BB	ECR → B, ECR → BB, ECR → ECU
H7	PL → FDS, PL → BB, FCR → PL	ECR → B, ECR → BB, ED → BB
H8	PL → FDS, FCR → BB	ECR → B, ECR → ECU
H9	PL → BB, PL → ECU, FDS → BB	ECR → B, ECR → BB, ED → BB
H10	PL → FDS, PL → BB, PL → ED	ECR → B, ECR → BB, ED → BB
H11	PL → BB, FDS → ECU	ECR → B, ECR → BB
H12	PL → ECR, PL → FDS, PL → BB	ECR → B, ECR → BB, ECR → ECU

### Results of closely related muscle pair direction coupling analysis

**Figure 3:**
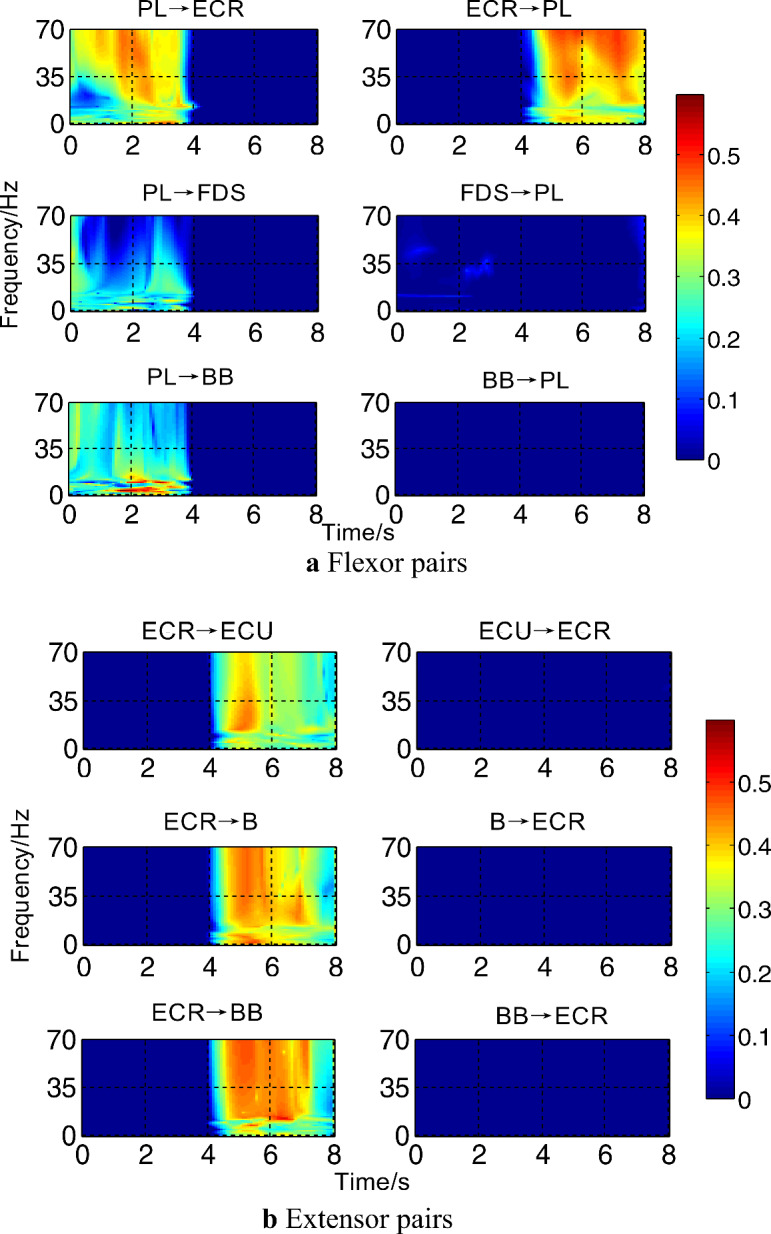
Results of significant TVPDC analysis for closely related muscle pairs.

To deeply explore the directional coupling properties of muscle pairs closely related to the continuous transition movements of wrist flexion and extension, the significant TVPDC values of flexor pairs (PL-ECR, PL-FDS, and PL-BB) and extensor pairs (ECR-ECU, ECR-BB, and ECR-BB) obtained by screening the same subjects were calculated, as shown in Fig. 3.

From Fig. 3a), the flexor pair PL→ECR had higher ${\text{DP}}_{xy} (k,f)$ values in the beta (15–35 Hz) and gamma (35–70 Hz) bands at the end of MC1 and the initial phase of WF, PL→FDS and PL→BB had higher ${\text{DP}}_{xy} (k,f)$ values in the theta (1–9 Hz) and alpha (8–15 Hz) bands at the WF phase, while ECR→PL had higher values in the beta and gamma bands at the WE phase, while the other two muscle pairs did not show any significant performance. From Fig. 3b), the overall coupling values of the extensor pairs in the MC2 phase are all higher than those in the WE phase, with ECR→ECU having higher A values in the beta band of the MC2 phase and ECR→B and ECR→BB having higher A values in the beta and gamma bands of the MC2 phase; in addition, all the above directional information transfer exhibits a unidirectional pattern, i.e., the reverse information transfer is almost zero.

**Figure 4:**
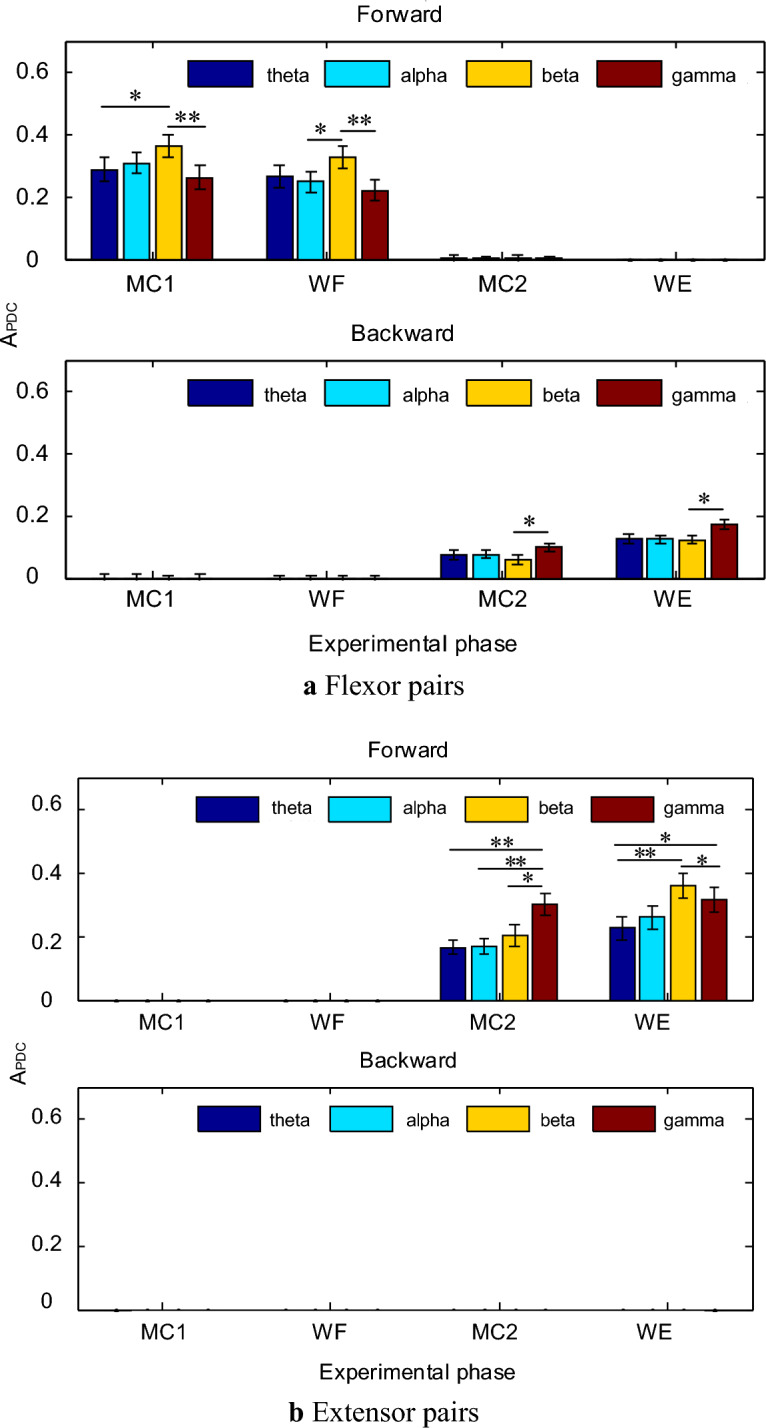
Means and variances of closely related muscles on significantly coherent area. (* indicate *p* < 0.05, ** indicate *p* < 0.01).

To analyze further the differences in the values of flexor and extensor pairs during different movement phases (MC1, WF, MC2 and WE) and within different frequency bands (theta(4–8 Hz), alpha(8–15 Hz), beta(15–30 Hz) and gamma(30–45 Hz)), the mean values of the closely related muscle pair values were calculated separately for 12 subjects and normalized, and the results are shown in Fig. 4.

From Fig. 4, the intensity of forward coupling was higher in the flexor pairs than in the extensor pairs at different movement phases under the overall angle. The $A_{{{\text{PDC}}}}$ value of forward coupling was larger in the beta band for the flexor pair than in the other bands during the MC1 and WF phases, as seen in Fig. 4a, and the $A_{{{\text{PDC}}}}$ value was smallest in the gamma band, and the differences were statistically significant (p<0.01) between both the beta and gamma bands; the flexor pairs had $A_{{{\text{PDC}}}}$ values of reverse coupling presence under MC2 and WE phases with a maximum in the gamma band, which was explained by the fact that the ECR, as the dominant muscle for wrist extension movements, showed information transmission in the ECR→PL direction. According to Fig. 4b, the $A_{{{\text{PDC}}}}$ value of forward coupling in the extensor pair was significantly higher in the WE phase than in the MC2 phase; the significant area within the gamma band was the largest under the MC2 phase, and the differences with the rest of the bands were all statistically significant; the $A_{{{\text{PDC}}}}$ in the beta band was the largest under the WE phase, followed by the gamma band, and the differences with theta and alpha bands were all statistically significant; the reverse coupling in the extensor pair did not show $A_{{{\text{PDC}}}}$ values in different movement phases, which was attributed to the absence of the dominant muscle in the extensor pair and the absence of reverse information transfer.

## Conclusion

To address the characteristics of intermuscular directional information transfer under continuous translational movements of wrist flexion and extension, the PDC analysis method and TVPDC analysis model were applied to the multi-channel intermuscular directional coupling characteristics of 12 healthy subjects. The experiment results showed that PL transmitted the most directional information to the rest of the muscles under wrist flexion, followed by FCR; ECR transmitted the most directional information to the rest of the muscles under wrist extension, and all of them had frequency band differences. In the significance analysis of the coupling strength of the muscle pairs closely related to the continuous wrist flexion–extension transition movements, the coupling strength of the flexor pairs was slightly higher than that of the extensor pairs. Specifically, the flexor pair had the largest positive coupling values in the beta band under MC1 and WF phases, the extensor pair had the largest coupling values in the gamma band under MC2 phase, and the largest coupling values in the beta band under WE phase. The above results fully illustrate that the TVPDC multichannel intermuscular directional coupling analysis model constructed in this paper can effectively portray the directional information transfer relationships in different frequency domains under different exercise phases, which provides some theoretical reference for exploring the control mechanism of CNS and the abnormal control mechanism in patients with motor dysfunction. The study of translational movements can provide a new perspective for kinematic research, and ergonomic research can help make the translational movements of robots more fluid and natural.

## Discussion

The multichannel intermuscular directional transmission characteristics under continuous wrist flexion and extension transitions were analyzed, and it was found that PL and ECR transmitted the most information to other muscles during wrist flexion and wrist extension movements in the time domain perspective, indicating that PL and ECR were the dominant motor muscles under wrist flexion and wrist extension movements, respectively; additionally, FCR also has a dominant role in the WF phase, while there are no remaining dominant muscles in the WE phase, which may be due to the fact that WE movements are more flexible in daily life, but WF movements require more muscles to execute the control information from the CNS^13^. There are also corresponding muscles that play a dominant role during the transition between wrist flexion and wrist extension, due to the fact that the CNS is also required to integrate relevant information to rapidly mobilize the corresponding muscles to support the transition movement during dynamic task execution^9^.

By screening, the muscle pairs closely related to the continuous transition movements of wrist flexion and extension were obtained, among which the flexor pair PL-ECR was more specific, as the dominant muscles under wrist flexion and wrist extension movements, transmitting control information to each other at different stages, respectively; PL→ECR has a higher coupling strength in the beta and gamma bands at the end of MC1 and under WF phase. The functions of the ECR under the WF phase from an anatomical point of view are similar to those of other responsive muscles, as one of the responsive muscles of the PL, and both the maintenance and higher information transfer functions of the wrist muscles during the completion of the steady-state task are related to the control mechanisms of the CNS within the beta and gamma bands; similarly, ECR→PL exhibits a similar interpretation of the information transfer law. The overall significant area under MC1 phase was greater than WF phase in the statistical analysis of flexor pairs, which may be due to the fact that during the initial phase of movement, the muscle requires more CNS control information to accomplish dynamic movements; most significant coupling characteristics in the beta band, which is due to the fact that the motion control information of the CNS is mainly in the beta band. In contrast, the coupling intensity within the gamma band is the lowest and significantly different, which is due to the fact that advanced information integration is temporarily not required at the beginning of the movement, manifesting as a lower coupling intensity within the gamma band.

## Methods

### Research subjects and experimental procedure

**Figure 5:**
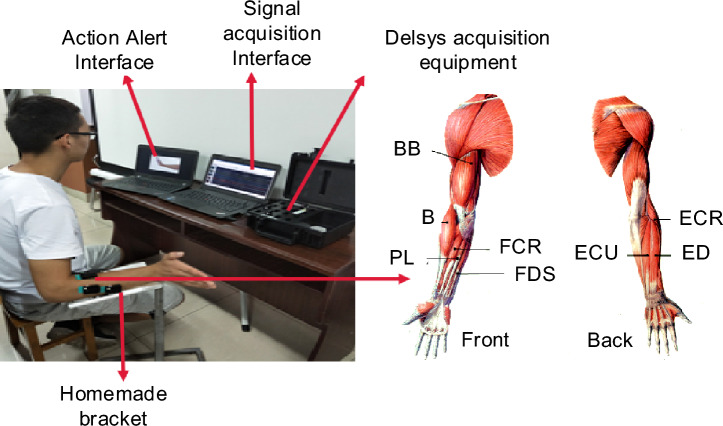
Wrist flexion extension continuous conversion exercise experiment.

As seen in Fig. 5, the upper limb wrist flexion–extension continuous transition movement test was designed, and the 8-channel sEMG associated with wrist flexion–extension movement(Biceps Brachii (BB), Brachioradialis (B), Radial Carpal Flexor (Flexor Carpi Radialis, FCR), Palmarislongus (PL), Radial Carpal Short Extensor (Extensor Carpi Radialis, ECR), Extensor Digitorum (ED), Extensor Carpi Ulnaris (ECU), and Flexor Digitorum Superficialis (FDS)) was collected simultaneously to study the change pattern of directional coupling between multiple muscles.

The data were obtained from 12 healthy adults (9 males and 3 females, age 25 ± 3 years), all subjects without upper limb motor dysfunction and all right-handed, who voluntarily participated in this data collection trial. For removing the influence of skin surface oil and dander on the signal acquisition, the skin of the signal acquisition site needs to be wiped with 75% medical alcohol concentration.

**Figure 6:**
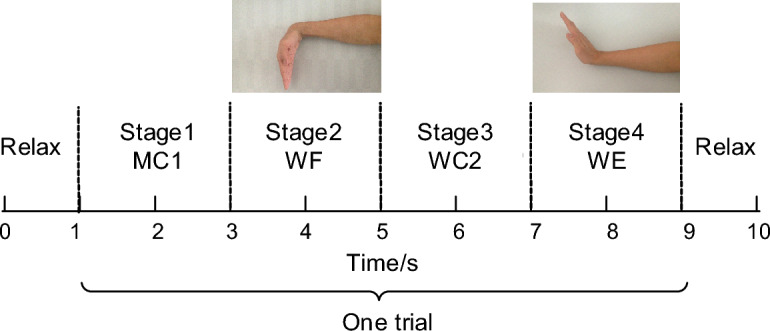
Experimental paradigm.

Before the test, the subject sat in front of a computer screen with the upper arm resting on a homemade brace used to support the upper arm to prevent fatigue and shoulder sway from affecting the test data, and there was no obstructive effect on wrist flexion and extension movements. The test process requires the subject to make the corresponding action according to the instructions of the computer screen, the test cycle is 10 s, the trial is 8 s, and the rest is 30 s after each cycle, totaling 10 cycles, the specific test paradigm is shown in Fig. 6.

### Electromyographic data pre-processing

**Figure 7:**
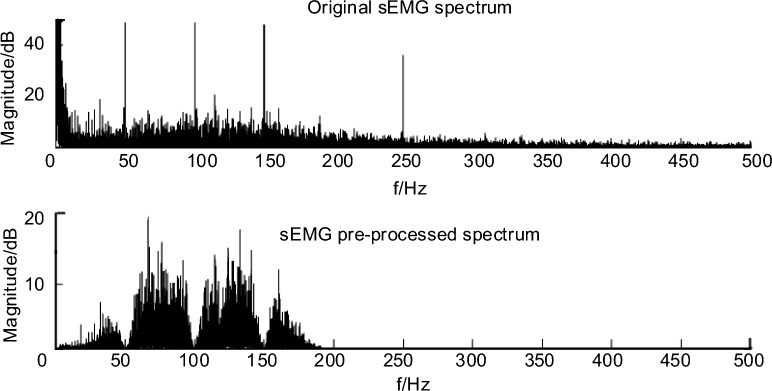
Spectrum of sEMG signal before and after preprocessing.

Since the sEMG signal is a nonstationary random signal, it is highly susceptible to interference from the external environment and acquisition equipment, and it needs to be pre-processed in order to obtain a higher quality signal for subsequent analysis. The results of sEMG pre-processing are shown in Fig. 7, which removes the baseline drift, industrial frequency and harmonic interference, and frequency band over-width, respectively.

Sliding average method to remove baseline drift.

The EMG is affected by the baseline stability performance of the acquisition device during the acquisition process, resulting in a slow unidirectional change over time, i.e., baseline drift. In this section, sliding average is used to remove the baseline drift.

The sliding average method is to select a suitable window size for a set of data, average all the abnormal data in the window as the center point abnormal value of the window, and then move the window by analogy until the average process of all data is completed.

Suppose the data *x(i),i* = *0,1,2,···, n* , the window length is *N(N* < *n/2)*, first calculate the average value of the data points of the first window *x*_*i*_(1), move the window backward in turn, the window step length is *k* , the data length of the fitted curve is *m* , satisfying *m* = *(n—N)/ p* and rounded. The final center point outlier fitting curve is* x*_*m*_*(i),i* = *0,1,2,···,m,* and finally the curve is upsampled as* x*_*m*_* (i), i* = *0,1,2,···,n*. The final median filtering result obtained is

$$ x_{l} \left( i \right) = x\left( i \right) - x_{m} \left( i \right),\quad \, i \, = \, 0,1,2,\cdot\cdot\cdot,n $$

Adaptive filter to remove 50 Hz industrial frequency and harmonic interference.

Since the acquisition equipment is powered by the daily 50 Hz AC power supply, it is accordingly represented in the EMG by the presence of 50 Hz sine waves and their harmonic spikes, i.e., industrial frequency interference. In this paper, the adaptive filter is used to remove the industrial frequency interference.

Butterworth bandpass filter to intercept the effective frequency band.

The effective frequency band of EMG is concentrated in the range of 0–250 Hz, which usually results in an overly wide EMG band due to the acquisition bandwidth of the acquisition equipment. Band-pass filtering method is a common method to obtain the effective frequency band in signal processing, and by setting reasonable low-pass and high-pass parameters, the retention of the effective frequency band of EMG signal can be achieved. In this paper, a fourth-order Butterworth bandpass filter is used to intercept the effective frequency band.

### Partial directional coherence analysis model

As GC can only analyze the causal characteristics between two channel signals, the evolved PDC can analyze the direct causality of multi-channel signals in the frequency domain at the same time, avoiding the indirect causal effects caused by other channels.The process of implementing PDC is as follows:

Set $x(t) = (x_{1} (t) \cdots x_{m} (t))^{{\text{H}}}$ is a smooth m-dimensional series with zero mean, then, the *p*-order AR model for x is

1 $$ \left( \begin{gathered} x_{{1}} (t) \hfill \\ \, \vdots \, \hfill \\ x_{m} (t) \hfill \\ \end{gathered} \right) = \sum\limits_{{r = {1}}}^{p} {{\text{A}}_{r} \left( \begin{gathered} x_{{1}} {(}t - r{)} \hfill \\ \, \vdots \hfill \\ x_{m} (t - r) \hfill \\ \end{gathered} \right)} + \left( \begin{gathered} \varepsilon_{{1}} (t) \hfill \\ \, \vdots \hfill \\ \varepsilon_{m} (t) \hfill \\ \end{gathered} \right) $$

in which, *p* is the order of the model,${\text{A}}_{r} (r = {1,2,} \cdots {,}p)$ is the coefficients matrix, $\varepsilon (t) = (\varepsilon_{{1}} (t) \cdots \varepsilon_{m} (t))$ is the white noise, and ${\text{A}}_{r}$ is the

2 $$ {\text{A}}_{r} = \left( \begin{gathered} a_{{{11}}} (r) a_{{{12}}} {(}r{) } \cdots \, \cdots \, a_{{{1}m}} (r) \hfill \\ \, \vdots \, \vdots \, \vdots \, \vdots \, \vdots \hfill \\ \, \vdots \, \vdots \, \vdots \, a_{ij} (r) \vdots \hfill \\ \, \vdots \, \vdots \, \vdots \, \vdots \, \vdots \hfill \\ a_{{m{1}}} (r) \cdots \, \cdots \, \cdots \, a_{mm} (r) \hfill \\ \end{gathered} \right) $$

where $a_{ij} (r)$ is the causal coefficient between variable $i$ and variable $j$. Performing the Fourier transform on both sides of Eq. (1) yields:

3 $$  {\bar{\text{{A}}}} = \left[ {\begin{array}{*{20}c}    {X_{1} \left( f \right)}  \\     \vdots   \\    {X_{m} \left( f \right)}  \\   \end{array} } \right] = \left[ {\begin{array}{*{20}c}    {E_{1} \left( f \right)}  \\     \vdots   \\    {E_{m} \left( f \right)}  \\   \end{array} } \right] $$

whereby $X\left( f \right)$ and $E\left( f \right)$ are the frequency domain representations of $x\left( t \right)$ and $\varepsilon\left( t \right)$, respectively. ${\bar{\mathrm{{A}}}}\left( f \right)$ is the coefficient matrix of $X\left( f \right)$_:_

4 $$ {\overline{\text{A}}}\left( f \right) = {\text{I}} - {\text{A}}\left( f \right) = {\text{I}} - \sum\limits_{{{\text{r}} = 1}}^{{\text{p}}} {{\text{A}}_{{\text{r}}} e^{ - j2\pi rf} } $$

whereas I is the M-dimensional unit matrix, ${\text{A}}(f)$ is the matrix of model coefficients after the Fourier transform of Eq. (1), and f is the frequency resolution, $\mathop {\text{A}}\limits^{\_}_{ij} (f)$ a between the $i$-th variable and the $j$-th variable can be defined as:

5 $$ \mathop {\text{A}}\limits^{\_}_{ij} (f) = \left\{ \begin{gathered} {1} - \sum\nolimits_{{r = {1}}}^{p} {a_{ij} (r)e^{{ - j{2}\pi rf}} ,i = j} \hfill \\ - \sum\nolimits_{{r = {1}}}^{p} {a_{ij} (r)e^{{ - j{2}\pi rf}} ,i \ne j} \hfill \\ \end{gathered} \right. $$

the PDC value of the $i$ th variable to the $j$ th variable is then to be defined as:

6 $$ {\text{PDC}}_{ij} \left( f \right) = \frac{{\left| {{\overline{\text{A}}}_{ij} (f)} \right|}}{{\sqrt {\overline{a}_{j}^{{\text{H}}} (f)\overline{a}{}_{j}(f)} }} $$

The following normalized parameter conditions are satisfied:

7 $$ {0} \le \left| {{\text{PDC}}_{ij} (f)^{2} } \right| \le {1,}\sum\limits_{i = 1}^{q} {\left| {{\text{PDC}}_{ij} (f)} \right|}^{2} = 1 $$

Similarly, the PDC value of the ith variable to the jth variable is defined as ${\text{PDC}}_{ji} (f)$.

For the purpose of describing the test results for each subject, the mean PDC values for 10 replicate trials were calculated for each subject in this paper:

8 $$ \overline{{{\text{PDC}}}} (f) = \frac{{1}}{{{10}}}\sum\limits_{{n = {1}}}^{{{10}}} {{\text{PDC}}_{n} (f)} $$

Here, ${\text{PDC}}_{n} (f)$ is ${\text{PDC}}_{ij} (f)$. All ${\text{PDC}}_{ij} (f)$'s mentioned below represent mean values.

### Time-varying partial directional coherence analysis model

Suppose the EMG signals of the two channels are $x(t)$ and $y(t)$, $t = {1,2,} \cdots {,}n$. Among them, $n$ is the total length of the signal, and the data are first divided into $K$ equal-length data segments using the unit energy Hamming window $w(t)$. And then the short-time Fourier transform within the corresponding window is as follows.

9 $$ X_{k} (f) = \frac{{1}}{{\text{T}}}x_{{1}} (t)w(t)e^{{ - j{2}\frac{{\uppi }}{{\text{T}}}f}} ,(k = {1,2,} \cdots {,}K) $$

10 $$ Y_{k} \left( f \right) = \frac{{1}}{{\text{T}}}y_{{1}} \left( t \right)w\left( t \right)e^{{ - j{2}\frac{{\uppi }}{{\text{T}}}f}} ,\left( {k = {1,2,} \cdots {,}K} \right) $$

In this case, $X_{{\text{k}}} (f)$ and $Y_{k} (f)$ are the time–frequency domain representations of $x(t)$ and $y(t)$, respectively, $x_{{1}} (t) = x\left[ {k{\text{(T}} - s{)} + t} \right]$ and $y_{{1}} (t) = y\left[ {k{\text{(T}} - s{)} + t} \right]$ are the two-channel time-domain signals under the $k$ th window, $T$ is the length of the window function $w(t)$, and $s$ is the number of overlapping samples. In this paper, it is set to T = 500 and s = 450.

Then, combining with Eq. (6), the time-varying partial directional coherence value is defined as:

11 $$ {\text{TVPDC}}_{ij} \left( {k,f} \right) = \frac{{{\overline{\text{A}}}_{ij} \left( {k,f} \right)}}{{\sqrt {\overline{a}_{j}^{{\text{H}}} \left( {k,f} \right)a_{j} \left( {k,f} \right)} }} $$

and where $a_{j} (k{,}f)$ is the $j$-th column of $\mathop {\text{A}}\limits^{\_} (k{,}f)$, $k$ is the number of windows, and $f$ is the frequency. Similarly, the following normalization conditions are satisfied.

12 $$ {0} \le \left| {{\text{TVPDC}}_{ij} {(}k,f{)}} \right|^{{2}} \le {1,}\sum\nolimits_{i = 1}^{q} {\left| {{\text{TVPDC}}_{ij} (k,f)} \right|^{2} = {1}} $$

which satisfies ${1} \le j \le q$, a value between 0 and 1 characterizes the strength of the directional coupling of the two channel EMG signals $x(t)$ and $y(t)$ at frequency $f$.

In the same way, to facilitate the description of the test results for each subject, the mean TVPDC values for 10 replicate trials were calculated for each subject in this paper as follows:

13 $$ \overline{{{\text{TVPDC}}}} (k{,}f) = \frac{{1}}{{{10}}}\sum\limits_{{n = {1}}}^{{{10}}} {{\text{TVPDC}}_{n} (k{,}f)} $$

In which, ${\text{TVPDC}}_{n} \left( {k,f} \right)$ is ${\text{TVPDC}}_{ij} \left( {k,f} \right)$. All ${\text{TVPDC}}_{ij} \left( {k,f} \right)$'s mentioned below represent the mean value.

To further characterize the significance of the ${\text{TVPDC}}_{ij} (k,f)$ values in the time–frequency range, the proxy data method was used to randomize the phase of the original data to obtain the proxy data ${\text{TVPDC}}^{\prime}_{ij} (k,f)$ without changing the time-domain amplitude and frequency-domain power spectrum values.

14 $$ {\text{DP}}_{ij} (k,f) = {\text{TVPDC}}_{ij} (k,f) - {\text{TVPDC}}^{\prime}_{ij} (k,f) $$

The ${\text{DP}}_{ij} (k{,}f)$ is the proxy data for 10 sets of repeated trials for each subject. If a value of ${\text{DP}}_{ij} (k{,}f)$ is positive, it indicates that the TVPDC is significant within that time–frequency point; if ${\text{DP}}_{ij} (k{,}f)$ is negative, it is considered to be significant to zero.

Moreover, this paper defines the significantly biased directional coherence area metric:

15 $$ A_{{{\text{PDC}}}} = \sum\limits_{{k = t_{1} }}^{{t_{2} }} {{\Delta }k} \sum\limits_{{f = f_{1} }}^{{f_{2} }} {{\Delta }f{\text{(DP}}_{ij} (k{,}f))} $$

${\Delta }k$ and ${\Delta }f$ are the time resolution and frequency resolution, respectively. The larger the $A_{{{\text{PDC}}}}$ value, the greater the strength of the directional coupling in that time–frequency range.

### Ethics approval

Approval was obtained from the ethics committee of Yan Shan University. The procedures used in this study adhere to the tenets of the Declaration of Helsinki. Demonstrate that all subjects and/or their legal guardians consent to the release of identifying information/images in online open access publications.

### Consent to participate

Informed consent was obtained from all individual participants included in the study.

## Data Availability

The datasets generated during and/or analysed during the current study are available from the corresponding author on reasonable request..
